# Supplementary material for: Spatial microenvironments tune immune response dynamics in the Drosophila larval fat body
Source: PLoS Genet. 2026 Feb 3;22(2):e1012029. doi: 10.1371/journal.pgen.1012029 (PMC12885378; doi:10.1371/journal.pgen.1012029)
Supplement: Attachment — Submitted filename: dpt_paper_plos_genetics_response_to_reviewers_1.pdf [file pgen.1012029.s028.pdf]

Please reply to:

Brandon Schlomann
University of California, Berkeley
Berkeley, CA 94720-3200
bschloma@berkeley.edu

July 18, 2025

Dear Editor,

Thank you for your assessment of our manuscript, “Spatial microenvironments tune immune response dynamics in the *Drosophila* larval fat body”. Below is a response to each comment raised by the reviewers, which accompanies our resubmission. Our paper uses a novel imaging technique to investigate repeatable spatial patterns of antimicrobial peptide expression in a key fly tissue, the fat body. The central issue raised by all reviewers was that the paper lacked mechanistic insight into the observed spatial pattern, with our experiments on the role of hemolymph flow unsatisfying, given their overall negative result. Therefore, we have significantly updated the paper, performing a series of genetic overexpression experiments that (1) definitively prove that the spatial pattern of immune activity is an intrinsic property of the fat body and is independent of infection and (2) identify that the rate-limiting step of antimicrobial peptide production is downstream of peptidoglycan sensing. These results are included in a new main text figure and Results section. In addition, at the request of Reviewer #1, we extended our findings to multiple new bacterial species. These additions to the paper are accompanied by text edits that clarify and streamline the narrative. Examples include refocusing the introduction on past work on fat body heterogeneity, shortening the description of the bacterial localization measurements, and simplifying the spatial transcriptomics analysis, focusing it on known immunity genes. In sum, we believe we have adequately addressed all reviewer concerns.

We look forward to hearing from you,

Brandon Schlomann

Detailed response to reviewers:

Reviewer #1: This study investigates the regionalized expression of antimicrobial peptide (AMP) genes along the fat body of Drosophila larvae. This work is particularly significant as the fat body has traditionally been considered a homogeneous organ by most researchers in the field. The authors convincingly demonstrate that the anterior and posterior lobes of the fat body exhibit greater immune reactivity. They further identify genes differentially expressed along the fat body by leveraging a previous microarray dataset. The imaging approach employed here is innovative and provides fresh insights into how spatial microenvironments influence immune responses in Drosophila.
While the study is compelling, it would benefit from a more thorough exploration of the mechanisms underlying the

observed heterogeneity. Below, I outline several suggestions to strengthen the manuscript.

Major Recommendations (Easily Addressable)
1. Infection Model Expansion:
It would be valuable to analyze the response of the fat body to oral bacterial infections, such as Erwinia carotovora carotovora 15 (Ecc15) (Basset et al., 2000). Given the proximity of the fat body to the gut, it is worth investigating whether this immune regionalization also occurs in the context of gut-associated infections.

As pointed out by Reviewer #2, this experiment has in fact already been done and was shown to produce a spatially heterogeneous DptA response that mirrors what we see with *E. coli* (Charroux and Royet, *PNAS* 2009). To more directly test if this expression pattern depends on the details of the infection, we instead opted for an overexpression strategy as suggested below, which revealed that this phenotype is independent of infection.

2. Diverse Pathogens:
All results are currently derived using a single bacterial strain, Escherichia coli. To strengthen the findings, I strongly recommend including other bacterial strains such as Pectobacterium carotovorum (Ecc15) or Providencia rettgeri, which produce varying amounts of peptidoglycan and may elicit distinct immune responses.

Reviewer #2 pointed us to data from Ecc15 (produced spatially heterogeneous DptA response). Therefore, we opted to expand the study to include *P. rettgeri* (produced spatially heterogeneous DptA response) and *E. faecalis* (produced uniform Drs and Mtk responses).

Recommendations to Further Strengthen the Study
3. Mechanistic Insights into Imd Pathway Regulation:
A plausible hypothesis for the observed differential expression of AMPs is that components of the Imd pathway, such as the PGRP-LC receptor, are unevenly distributed along the fat body.
1. Among the differentially expressed genes identified in the microarray, are there any encoding components of the Imd pathway?

We significantly revised our main spatial transcriptomics analysis to focus on known immunity genes. While primary components of the Imd pathway were not strong hits for spatially structured genes, we found other accessory candidates, including factors that regulate EcR signaling and components of the sumolyation pathway.

2. GFP-tagged versions of PGRP-LC (Neyen et al., Journal of Immunology, 2012) could be used to investigate whether the receptor's expression varies spatially (but they are weakly expressed).

We attempted to image this construct—expression levels were indeed low, barely above background in early L3, but to the extent that we could measure it, we saw no strong differences between fat body regions. We saw strong expression in wandering L3 fat bodies and the lymph gland. We described our observations in a new supplemental figure.

3. Analyzing Dpt-GFP expression under PGRP-LC overexpression (via heat shock or Gal4/Gal80 systems) could clarify whether the heterogeneity is due to changes in Imd pathway reactivity.

We did overexpression of both PGRP-LC.x, PGRP-LC.a, as well as a constitutively active form of Imd. All cases showed spatially heterogeneous DptA responses. The PGRP-LCs showed strong "U-shaped" expression patterns. The Imd construct also showed enhanced expression in the lateral fat body, but lacked expression in the dorsal-middle fat body. These experiments show that the different regions of the fat body do indeed have different levels of Imd pathway reactivity, and that binding of the peptidoglycan receptor is not the rate-limiting step of immune activation.

4. Experiments in PGRP-LB mutants (which exhibit reduced peptidoglycan cleavage) could also be informative in determining whether spatial variability persists when the Imd pathway ligand remains uncleaved.

Given the results of the PGRP-LC overexpression analysis, we concluded that peptidoglycan binding is not the rate-limiting step responsible for the spatial heterogeneity in AMP expression, and so deemed the PGRP-LB mutants not a priority. However, we are interested to explore how PGRP-LB regulates AMP expression dynamics in future work.

4. Improved Yeast Challenge Model:
The use of Saccharomyces cerevisiae as a challenge model is suboptimal due to its low immunogenicity. Consider using spores from Aspergillus or Beauveria species, which may elicit a more robust immune response.

We performed experiments using E. faecalis as a bacterial elicitor of Toll-dependent AMP responses. These results showed uniform Mtk and Drs expression patterns, which we describe in a new supplemental figure. We also added text clarifying that because of these mix results for Toll-dependent AMP responses, we focus the paper on Imd responses.

5. Expression Levels and AMP Dynamics:
The differential expression of AMPs (other than Diptericin) might be partly due to baseline expression levels. For instance, Drosocin and Defensin are expressed at lower levels compared to Drosomycin upon infection. This could be mentionned.

We are confused by this point. Does "baseline" mean without infection? We added text to this section emphasizing that Defensin was expressed at low levels and only in a subset of larvae.

6. Role of Abd-A in Immune Heterogeneity:
Exploring the potential involvement of Abd-A in shaping the spatial heterogeneity of the immune response could provide mechanistic insights on the regionalization.

We agree that this is an interesting next step, especially considering a recent preprint investigating spatial variations in metabolic behaviors across the fat body that are linked to the Bithorax Complex (Hemba-Waduge et al., bioRxiv 2024 doi:10.1101/2024.05.31.596851). We have added text to the discussion outlining this as important future work.

Minor Recommendation
7. Injection Method Clarification:
The mode of bacterial delivery (microinjection versus pricking) is an important methodological detail that should be explicitly mentioned in the Results section. Additionally, testing whether similar results are observed with pricking (a method that may minimally disrupt larvae) would enhance the robustness of the conclusions.

We added text in the first results section clarifying this. Since our overexpression experiments indicate that this phenotype is independent of the infection, we opted to not include addition experiments with the pricking method.

8. Contextual Background:
Including more information on the development and heterogeneity of the fat body in the Introduction or Discussion sections would provide valuable context for the reader and enhance the manuscript.

We significantly restructured the Introduction, focusing on what was known previously about fat body heterogeneity and leaving context about live imaging of immune responses in general for the Discussion.

Overall Assessment
This manuscript makes a significant contribution to our understanding of immune responses in Drosophila by challenging the traditional view of the fat body as a homogeneous organ. The imaging approach and findings are novel, but incorporating the above suggestions would strengthen the mechanistic depth and applicability of the work.

Reviewer #2: In this manuscript, the authors study the expression pattern of antimicrobial peptides in the fat body of Drosophila larvae injected with bacteria. They use state-of-the-art microscopy techniques and quantification tools. Analysis of the images obtained enabled them to conclude that Dipt A is expressed in a U-shaped expression pattern, with a maximum expression level in the anterior and posterior lobes of the fat body and a minimum in the center. Similar results were obtained using reporters for other AMPs under the control of either the IMD or the Toll pathways. A detailed analysis of the dynamics of AMP expression in cells led the authors to conclude that AMP expression is largely deterministic with spatially varying rates, rather than varying activation delays or stochastic dynamics. By analyzing the position of bacteria in larvae, the authors test the hypothesis that differential concentrations of bacteria in larvae explain the pattern of AMP expression. The results show that local bacterial concentration is not the main determinant of Dipt A expression patterns. Finally, the authors studied the role of the cardiac tube in the movement of bacteria in the larva and therefore potentially in the DiptA expression pattern, by blocking the circulation of hemolymph associated with cardiac movement. These experiments show that while the heart is indeed involved in bacterial movement, it is not involved in the establishment of the AMP expression pattern.
The manuscript is well written, with quality images and adhoc quantification of the phenotype studied.

Major issues

- The main conclusion of this manuscript is that DptA and AMPs in general are preferentially expressed in the anterior and posterior lobes of bacteria-infected larvae. Indeed, the second part of the manuscript, which seeks to determine the reasons for this expression pattern, is inconclusive. In fine, the manuscript remains very descriptive (of a already reported phenotype, see below) and fail short in providing on the mechanisms involved;

We agree with this assessment and added a series of new experiments that aimed to identify the underlying mechanism. We measured DptA expression patterns in response to overexpression of various Imd pathway components, in the absence of infection. Overexpression of peptidoglycan receptors produced a similar “U-shaped” expression pattern of DptA, demonstrating that this phenotype is independent of infection. Overexpression of a constitutively active Imd protein showed a more uniform, but still incomplete response. We complemented these genetic perturbations with imaging of fluorescently tagged PGRP-LC and Relish. While we don’t yet have a “smoking gun” for the molecular mechanism restricting DptA expression in the middle-dorsal fat body, we have placed the mechanism acting downstream of PGRP activation.

- The authors express surprise at this bi-modal expression pattern, which differs from what has been obtained and published with Dipt LacZ. However, it is undoubtedly the latter that does not reflect reality. Indeed, there are already images in the literature showing this U-shaped activation of DiptA in bacterially infected larvae. In PMID: 19482944, figure 4 shows photos of Dipt Cherry larvae infected with Ecc bacteria. The U-shape expression pattern is clearly visible. It is interesting to note that these larvae were infected by ingestion of Ecc bacteria and not by injection as in the present manuscript. However, it is accepted that orally ingested Ecc do not cross the gut epithelium and can induce fat body Dipt expression as a U-shape without being present in the hemolymph. In view of these results, the experiments presented in this manuscript testing the role of bacterial localization are not really relevant, since the same result can be obtained without contact between the bacteria and the fat body. Finally, I'd like to point out to the authors that AMPs can be induced independently of bacteria, by stress, and that this is also reflected in this U-shape expression. This suggests that the anterior and posterior lobes of the fat body have a heightened sensitivity to the rest of the fat body to activate AMPs, which is clearly independent of the local concentration of bacterial elicitor and corresponds rather to an intrinsic characteristic of the tissue potentially linked to their developmental history. It would be interesting to test the role of the hox genes that segment the larva in the acquisition of this characteristic.

We thank the reviewer for pointing out this extremely relevant reference. We have reframed the paper, removing language around our “discovery” of this spatial expression pattern, and emphasize more our characterization of its dynamics and underlying mechanisms using our novel imaging method. We have refocused the narrative, eliminating any suggestions of localized microbial signals causing the response to reflect our new data showing that the AMP expression pattern is independent of infection.

Reviewer #3: In this manuscript, Schlomann and colleagues present the intriguing observation that immune response to bacterial injection in the larval fruit fly fat body is spatially-structured. They test several hypotheses about the origins of this structure, concluding that certain regions may be primed to produce antimicrobial peptides.

The observation of the spatially-structured response is interesting and novel. The methods are presented in detail, and there was substantial methods development required for the work, especially for long-term live imaging of these samples. The authors do not have definitive evidence for the priming hypothesis, but the paper provides useful empirical

information on the open problem of the role of tissue structure in immune response. However, I do not think that the conclusion that activated regions have higher bacterial exposure due to blood flow is supported by the data, as presented. Additionally, I would like to see stronger analysis to support the claim that the patterning is not due directly to an uneven distribution of bacteria, and more generally on the relationship between bacterial exposure and AMP production.

Comments:

Bacterial concentration, heartbeat, and response: This section is confusing, and I do not believe the conclusions are supported by the data as presented. There are three separate questions here: 1. Does bacterial load in a particular region predict response, 2. Does heartbeat/blood transport determine the spatial distribution of bacteria; and 3. Does heartbeat affect the spatial response pattern. 1 and 3 are questions that bear directly on the causes of the spatial patterning, while 2 is primarily distracting. Further, I do not think that the conclusion the authors draw in answer to 2 is supported by Figure 4. In particular, the authors claim that, due to blood flow, planktonic bacteria occur preferentially at the anterior and posterior. However, from Figure 4, there appears to be barely any additional bacteria in the anterior in the presence of heartbeat; the spatial pattern in fact appears a bit stronger in the absence of heartbeat. Thus it would seem that the heartbeat actually has little effect on the spatial distribution of the bacteria. In fact, it seems like the main effect is that there are substantially more planktonic bacteria everywhere in the absence of heartbeat.

The authors could reorganize this section to clarify it. Additionally, I would like to see clearer analysis to support the claim that bacterial load does not determine the AMP response. This analysis would extend Figure S6, which appears to me to bear more on the central questions here than much of Figure 4. In particular, the authors could analyze the extent to which the observed variation in cellular activation can be explained by different densities of bacteria, given no knowledge of spatial location: how well does the best monotonic model mapping a bacterial density to a response amplitude do in predicting responses? How does this compare to a model based only on physical location? Additionally, given the heterogeneity from larva to larva, the authors should do this analysis at the single-larval level if possible. It would also be useful to see maps of DptA-GFP fluorescence distribution and bacterial distribution for individuals. These analyses would add substantially to demonstrating that bacterial distribution cannot explain the observed spatial patterning.

We agree with this assessment and have reframed the paper. We performed a series of experiments overexpressing immune signaling pathway components that demonstrate that the spatial pattern of AMP response is in fact independent of infection. The section on bacterial distribution and heartbeat knock out has been streamlined and reframed clearly as a negative result.]

Additional comments on this section: 1. Does the additional planktonic bacterial load in the absence of heartbeat lead to higher overall DptA-GFP response?

Thank you for the question. The DptA-GFP response is actually 3x lower in animals without a heartbeat, despite the increased bacterial load. However, these animals also develop more slowly, so we are hesitant to read too much into this difference, as it could be caused by a multitude of physiological factors.

2. I think the presentation would be clearer if this section had a first figure relating bacterial density to DptA-GFP response, and a second figure showing that knocking out heartbeat does not erase the spatial pattern.

We have re-ordered this figure, but in a slightly different way that we hope is more clear.

Based on these considerations, I do not think that the sentences in the abstract 'This pattern correlates with microbial localization via blood flow but is not caused by it: loss of heartbeat suppresses microbial transport but leaves the expression pattern unchanged. This result suggests that regions of the tissue most likely to encounter microbes via blood flow are primed to produce antimicrobials' are supported.

We have removed this sentence and more broadly re-wrote the abstract to reflect the new results from overexpression experiments.
