# Supplementary material for: Spatial microenvironments tune immune response dynamics in the Drosophila larval fat body
Source: PLoS Genet. 2026 Feb 3;22(2):e1012029. doi: 10.1371/journal.pgen.1012029 (PMC12885378; doi:10.1371/journal.pgen.1012029)
Supplement: Attachment — Submitted filename: dpt_paper_plos_genetics_response_to_reviewers_2.pdf [file pgen.1012029.s029.pdf]

UNIVERSITY OF CALIFORNIA, BERKELEY

BERKELEY • DAVIS • IRVINE • LOS ANGELES • RIVERSIDE • SAN DIEGO • SAN FRANCISCO SANTA BARBARA • SANTA CRUZ

Please reply to:

Brandon Schlomann
University of California, Berkeley
Berkeley, CA 94720-3200
bschloma@berkeley.edu

October 10, 2025

Dear Editor,

Thank you for your assessment of our revised manuscript, “Spatial microenvironments tune immune response dynamics in the *Drosophila* larval fat body”. We are thrilled that Reviewers #1 and #3 approve our revisions and greenlight the manuscript. Following correspondence with the Editorial Team, we believe we have addressed the remaining concerns of Reviewer #2 (detailed below) by (i) emphasizing the quantitative imaging aspect of our paper as the key innovation and (ii) softening all claims of identifying the underlying mechanisms of heterogeneous immune activity in the fat body. We now more plainly state that uncovering the precise molecular mechanisms involved requires additional work. We believe these changes make the manuscript a more transparent representation of our work, which presents the first single-cell measurements of antimicrobial peptide expression dynamics during infection in live flies and uses this quantitative readout together with genetic perturbations to conclusively reveal the presence of defined immune microenvironments within the larval fat body.

We look forward to hearing from you,

Brandon Schlomann

Detailed response to reviewers:
Reviewer #1: The article has been improved. I recommend its publication. They have addressed most of my recommendation.

Author response: Thank you for the useful suggestions, we are happy to have addressed your recommendations.

Reviewer #2: This manuscript describes a phenomenon already reported whereby, in larvae infected with bacteria, the transcriptional activation of diptericin, a readout of the activation of one of the NF-kB IMD pathways, is uneven and more pronounced in the anterior and posterior ends of the fat body lobes. The added value of this study lies in its quantitative aspect, which provides a more accurate assessment of the phenomenon described. My main criticism was that this study remained highly descriptive, without

demonstrated mechanisms to understand the cellular and molecular explanations for this differential expression and its function. In the revised version, the authors show that overexpression of a constitutive version of PGRP-LC mimics the injection of E. coli, suggesting that the central part of the fat body is not competent to activate the IMD pathway. However, we still do not know the reasons for this incompetence. Surprisingly, the mCD8-GFP protein, which has nothing to do with the IMD pathway, also has a U-shaped pattern. Furthermore, the use of E faecalis as an inducer shows that the Toll pathway is activated throughout the fat body. My impression after reading this new version of the manuscript remains the same. Through quantification, they describe a heterogeneity of activation of the IMD pathway in the fat body (already described). There is no data available to enable us to assess which cellular and molecular mechanisms (i) that explain this heterogeneity, (ii) that explain why what is true for the IMD pathway is not for the Toll pathway, and (iii) determine the role of this phenomenon in the immune response or in the biology of the larva more generally.

Author response: Thank you for your comments. We agree that the cellular and molecular mechanisms behind the heterogeneous AMP response remain unclear and are excited to pursue them in future work. However, we feel that even without these mechanisms identified, the paper stands on its own, contributing the first measurements of single-cell AMP expression dynamics in live flies, rigorous quantitative characterization of AMP heterogeneity within the fat body, and conclusive evidence that the heterogeneity is independent of infection. Therefore, to address your concerns, we have made several text edits throughout the Abstract, Introduction, and Discussion that (i) re-emphasize the quantitative imaging aspect of our work as the key innovation, and (ii) soften all claims of identifying the molecular mechanism underlying the heterogeneity (e.g., we removed the word "mechanism" from the abstract) and plainly state that more work is required to identify these mechanisms. We believe these changes make the manuscript a more transparent reflection of our work.

Regarding your comment about the mCD8 expression pattern—we disagree with your interpretation that this expression pattern is "U-shaped". The anterior does indeed show a small peak that is ~1.3x larger than the rest of the fat body, which may be indicative of some generic differences in gene expression in this region. But this effect size is tiny compared to the DptA pattern, for which, the anterior is ~14x larger than the middle, and the posterior is ~40x larger. As described in the Fig. 5 caption, the "dips" in expression adjacent to the anterior and posterior regions in the mCD8-mCherry pattern are due to the anatomy of the fat body—there simply are not many fat body cells in these regions. We have added text in the Results section that clarifies this point.

Reviewer #3: The authors have strengthened and clarified the manuscript; I have no further comments/concerns.

Author response: Thank you for the useful suggestions, we are happy to have addressed your concerns.
